# The tyrosine kinase v‐Src modifies cytotoxicities of anticancer drugs targeting cell division

**DOI:** 10.1111/jcmm.16270

**Published:** 2021-01-19

**Authors:** Ryuzaburo Yuki, Mari Hagino, Sachi Ueno, Takahisa Kuga, Youhei Saito, Yasunori Fukumoto, Noritaka Yamaguchi, Naoto Yamaguchi, Yuji Nakayama

**Affiliations:** ^1^ Department of Biochemistry and Molecular Biology Kyoto Pharmaceutical University Kyoto Japan; ^2^ Department of Molecular Cell Biology, Graduate School of Pharmaceutical Sciences Chiba University Chiba Japan

**Keywords:** anti‐mitotic drugs, cancer cell survival, microtubule‐targeting agents, mitotic slippage, v‐Src

## Abstract

v‐Src oncogene causes cell transformation through its strong tyrosine kinase activity. We have revealed that v‐Src‐mediated cell transformation occurs at a low frequency and it is attributed to mitotic abnormalities‐mediated chromosome instability. v‐Src directly phosphorylates Tyr‐15 of cyclin‐dependent kinase 1 (CDK1), thereby causing mitotic slippage and reduction in Eg5 inhibitor cytotoxicity. However, it is not clear whether v‐Src modifies cytotoxicities of the other anticancer drugs targeting cell division. In this study, we found that v‐Src restores cancer cell viability reduced by various microtubule‐targeting agents (MTAs), although v‐Src does not alter cytotoxicity of DNA‐damaging anticancer drugs. v‐Src causes mitotic slippage of MTAs‐treated cells, consequently generating proliferating tetraploid cells. We further demonstrate that v‐Src also restores cell viability reduced by a polo‐like kinase 1 (PLK1) inhibitor. Interestingly, treatment with Aurora kinase inhibitor strongly induces cell death when cells express v‐Src. These results suggest that the v‐Src modifies cytotoxicities of anticancer drugs targeting cell division. Highly activated Src‐induced resistance to MTAs through mitotic slippage might have a risk to enhance the malignancy of cancer cells through the increase in chromosome instability upon chemotherapy using MTAs.

## 1 INTRODUCTION

The non‐receptor‐type tyrosine kinase v‐Src is the oncogene first identified from Rous sarcoma virus.^1^ v‐Src shows a high level of kinase activity compared with cellular counterpart c‐Src, as the negative regulatory region at the C‐terminus of c‐Src is lacking in v‐Src.^2^, ^3^, ^4^ Constitutively activated v‐Src leads to anchorage‐independent cell growth and cell transformation through the following cellular alterations^5^, ^6^: (i) actin cytoskeleton reorganization, (ii) loss of contact inhibition ability along with lack of cell–cell interaction and (iii) growth signal promotion. v‐Src promotes cell growth signals such as activation of MAPK/ERK pathway and CDK‐cyclin complexes. However, we showed that the inducible expression of v‐Src inhibits cell proliferation along with up‐regulation of the CDK inhibitor p21.^7^, ^8^ Given that v‐Src causes mitotic defects such as chromosome bridge formation and brings about colony formation at a low frequency, v‐Src‐mediated cell transformation may be attributed to stochastic results of chromosomal abnormalities.^7^, ^8^, ^9^ Among the heterogeneous cell population generated by chromosomal abnormalities, survived cells from v‐Src‐caused inhibition of proliferation may contribute to proliferate to malignant cancers.

Recently, we found a new event of mitotic defects in v‐Src‐expressing cells^10^: v‐Src induces mitotic slippage by directly phosphorylating CDK1 in mitosis, resulting in CDK1 inactivation and premature mitotic exit. Even when cells are treated with S‐trityl‐L‐cysteine (STLC), an inhibitor of the kinesin motor protein Eg5, and arrested at mitosis, v‐Src causes mitotic slippage and suppresses Eg5 inhibitor‐induced cytotoxic effects. It is known that oncogenes confer resistance to various anticancer drugs through several mechanisms, such as activation of the compensational pathway and genetic alternations of drug target proteins.^11^, ^12^ Oncogene‐induced mitotic slippage may be a way to develop resistance against anticancer drugs that target cell division in cancer cells.

In this study, we demonstrated that v‐Src suppressed the cytotoxicity of several MTAs that cause mitotic arrest, but not anticancer drugs that generate DNA damages. By time‐lapse imaging analysis, v‐Src caused mitotic slippage in MTAs‐treated mitotically arrested cells. Furthermore, we investigated whether v‐Src alters the effects of the inhibitors of important mitotic kinases such as PLK1 and Aurora kinases. v‐Src slightly suppressed the cytotoxicity of PLK1 inhibitor as well. Surprisingly, the Aurora kinase inhibitor drastically decreased the cell viability of v‐Src‐expressing cells.

## 2 MATERIALS AND METHODS

### 2.1 Cells

We previously generated HeLa S3 or HCT116 cells that can inducibly express v‐Src upon doxycycline (Dox) treatment (HeLa S3/v‐Src or HCT116/v‐Src)^7^ using v‐Src DNA from pcDNA3/v‐Src (gifted by Ohnishi).^13^ These cells were cultured in Dulbecco’s modified Eagle’s medium containing 5% fetal bovine serum with 20 mM HEPES–NaOH (pH 7.4) at 37ºC in 5% CO_2_.

### 2.2 Chemicals

Paclitaxel (169‐18611; FujiFilm Wako) and vincristine (87424; Nippon Kayaku, Japan), commonly used MTAs, were used at 0.1 µg/mL and 10 µM, respectively. The Eg5 inhibitor STLC (164739; MilliporeSigma, Burlington, MA) was used at 10 µM to activate the spindle assembly checkpoint (SAC). The monopolar spindle (MPS)‐1 inhibitor AZ3146 (11170; AdooQ BioScience, BioScience, Irvine, CA) was used at 2.5 µM to inactivate SAC by MPS1 inhibition. The PLK1 inhibitor BI2536 (A10134; AdooQ BioScience) was used at 50 or 200 nM. The Aurora kinase inhibitor ZM447439 (JS Research Chemicals Trading, Wedel, Germany) was used at 2–20 µM. The anticancer drug adriamycin (Sigma‐Aldrich, St. Louis, MO) was used at 10 or 1000 nM, and bleomycin (874234; Nippon Kayaku) was used at 5 or 100 µM.

### 2.3 Viability assay

The number of live cells per well was estimated using a Cell Counting Kit‐8 (Dojindo, Kumamoto, Japan) according to the manufacturer’s instructions as described previously.^14^ In brief, HeLa S3/v‐Src cells at 3.0 or 8.0 × 10^3^ cells/well in a 96‐well plate were cultured with or without inhibitors/anticancer drugs in the presence or absence of 2 ng/mL Dox for 24 hours. After washing these drugs out, the cells were further cultured for 2 days. The absorbance (450 nm) of reduced 2‐(2‐methoxy‐4‐nitrophenyl)‐3‐(4‐nitrophenyl)‐5‐(2,4‐isulfophenyl)‐2H‐tetrazolium, monosodium salt (WST‐8) was measured using an iMark Microplate Reader (Bio‐Rad).

### 2.4 Antibodies

The primary antibodies used for immunofluorescence (IF), immunoblotting (IB), and flow cytometry (FC) were as follows: mouse monoclonal anti‐γ‐tubulin (IF, 1:200; GTU‐88, T6557, Merck Millipore, Darmstadt, Germany), mouse monoclonal anti‐Src (IB, 1:200–500; GD11, Merck Millipore), rabbit polyclonal anti‐active Src (IB, 1:1000; pY416, #2101, Cell Signaling Technology, Danvers, MA), rabbit polyclonal anti‐cyclin B1 (FC, 1:250; sc‐752, Santa Cruz Biotechnology, Dallas, TX) and rat monoclonal anti‐α‐tubulin (IB, 1:2000; MCA78G, Bio‐Rad) antibodies. For IF, Alexa Fluor 555‐labelled donkey antimouse IgG antibody (1:1000; Life Technologies, Carlsbad, CA) was used. For IB, horseradish peroxidase‐conjugated antimouse (1:8000; 715‐035‐151, Jackson ImmunoResearch), anti‐rabbit (1:8000; 711‐035‐152, Jackson ImmunoResearch) and anti‐rat (1:8000; 712‐035‐153, Jackson ImmunoResearch) IgG antibodies were used. For FC, Alexa Fluor 488‐labelled donkey anti‐rabbit IgG antibody (1:1000; A21206, Life Technologies) was used.

### 2.5 Immunofluorescence microscopy

Immunofluorescence staining was performed as described previously.^14^ In brief, cells were cultured on coverslips and fixed with 100% methanol at −30ºC for 5 minutes. After permeabilizing and blocking with PBS(−) containing 0.1% saponin and 3% bovine serum albumin for 30 minutes, the cells were incubated with the primary antibody for 1 hour and subsequently with the secondary antibody for 1 hour along with 1 µM Hoechst 33342 for DNA staining. The fluorescence images were obtained using an IX‐83 fluorescence microscope (Olympus, Tokyo, Japan) equipped with a ×60 1.42 NA oil‐immersion objective lens (Olympus). The optical system included a U‐FUNA filter cube (360–370 nm excitation, 420–460 nm emission) and a U‐FRFP filter cube (535–555 nm excitation, 570–625 nm emission) to observe Hoechst 33342 and Alexa Fluor 555 fluorescence, respectively. The captured images were edited using Photoshop CC and Illustrator CC software (Adobe).

### 2.6 Flow cytometry

To collect dead and detached cells, floating cells in culture supernatants were collected and mixed with cells detached by trypsinization. For DNA and cyclin B1 staining in fixed cells, the cells were fixed with 70% ethanol at −30°C for 1 hour. After washing the cells with PBS(−) containing 3% calf serum plus 0.1% Triton X‐100, the cells were incubated at room temperature with anti‐cyclin B1 antibody and subsequently with Alexa Fluor 488‐labelled donkey anti‐rabbit IgG antibody in PBS(−) containing 3% calf serum plus 0.1% Triton X‐100 for 1 hour in the dark. Then, the cells were stained for DNA with 50 µg/ml propidium iodide (PI) plus 200 µg/mL RNase A at 37°C for 30 minutes in the dark. For DNA and Annexin V staining in unfixed cells, an Annexin V‐FITC Apoptosis Detection Kit (Nacalai Tesque, Kyoto, Japan) was used according to the manufacturer’s instructions. In brief, collected cells were incubated at room temperature with Annexin V‐FITC conjugate and PI in Annexin V binding solution for 15 minutes in the dark. Immediately, the stained cells were analysed using a flow cytometer equipped with a 488 nm solid‐state blue laser and a 640 nm diode red laser (BD Accuri C6 Plus; BD Biosciences, San Jose, CA). Debris was excluded based on the forward and side scatter profiles. FlowJo software (Tree Star, Ashland, OR) was used for data analysis and plot.

### 2.7 Time‐lapse imaging

Time‐lapse imaging was performed as described previously.^15^, ^16^ In brief, HeLa S3/v‐Src and HCT116/v‐Src cells were cultured with 2 ng/mL and 1 ng/mL Dox, respectively, for 11 hours, in the presence of 0.1 µM Hoechst 33342 to stain DNA during the last 1 hour. Then, time‐lapse imaging was performed using a high‐content imaging system (Operetta, PerkinElmer Life Sciences, Waltham, MA) at 37°C in 5% CO_2_.

### 2.8 Western blotting

Whole cell lysates were prepared by solubilizing cells in sodium dodecyl sulphate (SDS) sample buffer containing phosphatase inhibitors (50 mM NaF, 20 mM β‐glycerophosphate and 10 mM Na_3_VO_4_). The cell lysates were subjected to SDS‐polyacrylamide gel electrophoresis (PAGE) and electrotransferred onto polyvinylidene difluoride membranes (PVDF; Pall Corporation, Port Washington, NY). After blocking with Blocking One (Nacalai Tesque) at room temperature for 30 minutes, the membranes were incubated with the antibodies, which were diluted with Tween 20‐containing Tris‐buffered saline [20 mM Tris–HCl (pH 7.5), 137 mM NaCl and 0.1% Tween 20] containing 5% Blocking One. Clarity (Bio‐Rad) was used as the chemiluminescence substrate. A ChemiDoc XRSplus image analyser (Bio‐Rad) was used for the chemiluminescence detection and band intensity analysis.

### 2.9 Statistics

Statistical differences between two datasets were analysed using Student's *t* test after analysis of variance by F test. Statistical differences among more than two datasets were analysed using one‐way ANOVA with Tukey's post hoc test or Welch's ANOVA with Games–Howell post hoc test, based on their variance that was analysed using Bartlett's test. Statistical analysis was performed using Microsoft Excel program (Microsoft, Redmond, WA), EZR software (v. 1.41; Saitama Medical Center, Jichi Medical University, Saitama, Japan)^17^ and R software (v.3.4.3; R Foundation for Statistical Computing, Vienna, Austria).

## 3 RESULTS

### 3.1 Suppressive effects of v‐Src on the cytotoxicity of microtubule‐targeting agents

**FIGURE 1:**
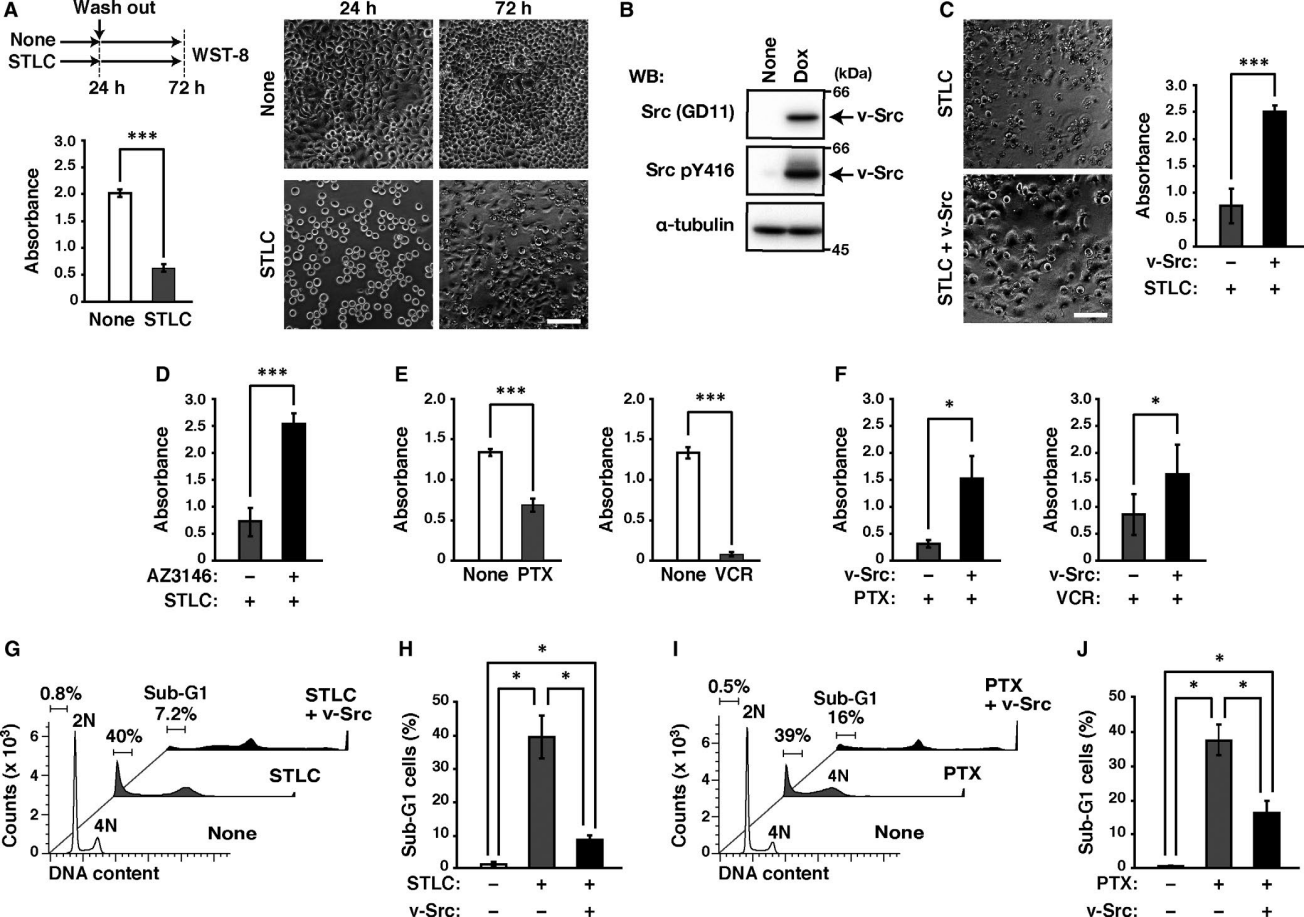
v‐Src suppresses microtubule‐targeting agents–induced reduction in cancer cell viability. A, (Upper left) Schematic depiction of drug treatment. HeLa S3/v‐Src cells were cultured with or without 20 µM S‐trityl‐L‐cysteine (STLC) for 24 hours. After washing STLC out, these cells were further cultured without any drug for 48 hours. (Right) Phase‐contrast images were obtained just after STLC treatment (24 hours) and 48 hours after STLC removal (72 hours). Scale bar, 50 µm. B, HeLa S3/v‐Src cells were cultured with or without 2 ng/mL doxycycline (Dox) for 24 hours. Whole cell lysates were analysed by Western blotting using anti‐Src, anti‐active Src (Src pY416) and anti‐α‐tubulin antibodies. Full blots are shown in Figure S2A. C, HeLa S3/v‐Src cells were cultured with or without 2 ng/mL Dox in the presence of 20 µM STLC, as shown in A. (Left) Phase‐contrast images were obtained 48 hours after indicated drugs removal. Scale bar, 50 µm. D, HeLa S3/v‐Src cells were cultured with or without 2.5 µM AZ3146 in the presence of 20 µM STLC, as shown in A. E, HeLa S3/v‐Src cells were cultured with or without 0.1 µg/mL paclitaxel (PTX) (left) or 10 µM vincristine (VCR) (right), as shown in A. F, HeLa S3/v‐Src cells were cultured with or without 2 ng/mL Dox in the presence of 0.1 µg/mL PTX (left) or 10 µM VCR (right), as shown in A. A, C–F, HeLa S3/v‐Src cells were plated at a density of 8.0 × 10^3^ cells/well of a 96‐well plate. Cell viability was determined by WST‐8 assay 48 hours after removal of indicated drugs. Graphs represent the mean ± SD of three independent experiments. Asterisks indicate significant differences (Student's *t* test, ^*^ *P* < .05; ^***^ *P* < .001). G–J, HeLa S3/v‐Src cells were cultured with or without 2 ng/mL Dox in the presence or absence of 20 µM STLC (G, H) or 0.1 µg/mL PTX (I, J) for 24 hours. Then, the cells were washed and further cultured without any drug for 72 hours. The cells were fixed, stained with propidium iodide (PI) (DNA staining) and subsequently analysed by flow cytometry. DNA histograms and the ratios of Sub‐G1 cells are shown, and each plot represents 40,000 cells. The ratios of Sub‐G1 cells are plotted, and graphs represent the mean ± SD of three independent experiments. Asterisks indicate significant differences (Games–Howell test, ^*^ *P* < .05)

Anti‐mitotic drugs have been commonly used as anticancer drugs. One class of popular anti‐mitotic drugs are MTAs, which prolongs mitosis by activating the spindle assembly checkpoint (SAC) and causes mitotic cell death within prolonged mitosis. Recently, we showed that v‐Src induces mitotic slippage by inactivating CDK1 in mitosis by directly phosphorylating CDK1 at Tyr‐15.^10^ The 1‐day treatment of STLC, an inhibitor of the mitotic motor kinesin Eg5, sufficiently accumulated mitotic cells, which were detached from surrounding cells and rounded up. The following 2‐day culture reduced the cell number, even though the drugs were washed out (Figure 1A). The number of cells survived from STLC treatment was drastically increased by v‐Src whose expression was induced by Dox treatment (Figure 1B,C), as previously reported.^10^ This indicates that v‐Src reduces STLC cytotoxicity (Figure 1C).

SAC components are recruited to microtubule‐unattached kinetochores through kinetochore‐associated kinase MPS1‐mediated phosphorylation, and SAC stops chromosome segregation while all kinetochores are not properly attached to microtubules. Therefore, MPS1 inhibition inactivates SAC, leading to premature mitotic progression. Considering that prolonged arrest in the prometaphase‐like state through SAC activation is required for the STLC cytotoxicity, it is hypothesized that v‐Src ended this arrest by inducing mitotic slippage and affected the STLC cytotoxicity. Based on this, it is expected that agents capable of inhibiting SAC and allowing cells to begin anaphase onset would reduce the cytotoxicity of STLC. Indeed, the MPS1 inhibitor AZ3146 treatment restored the cell viability reduced by STLC as v‐Src did (Figure 1D), confirming that v‐Src reduces the STLC cytotoxicity through SAC inactivation.

Given that resolution of mitotic arrest by SAC inactivation may attenuate the cytotoxic effect of MTAs,^18^ we here investigated whether v‐Src suppressed sensitivity to other MTAs than STLC. Paclitaxel and vincristine, both clinically used MTAs, reduced cell viability in the absence of v‐Src expression (Figure 1E). Expectedly, v‐Src partially prevented paclitaxel‐ and vincristine‐induced reduction in cell viability (Figure 1F), suggesting that v‐Src confers the resistance to MTAs not only to STLC.

Furthermore, to examine whether v‐Src affected cell death caused by MTAs, we analysed the population of Sub‐G1 cells, including dead DNA‐fragmented cells, by flow cytometry. Although STLC or paclitaxel treatment strongly increased the ratios of Sub‐G1 cells, these ratios were decreased by v‐Src expression in STLC‐ or paclitaxel‐treated cells (Figure 1G‐J). These results suggest that v‐Src suppresses the cytotoxicity of MTAs.

### 3.2 Effect of v‐Src on the cytotoxicity of adriamycin and bleomycin

**FIGURE 2:**
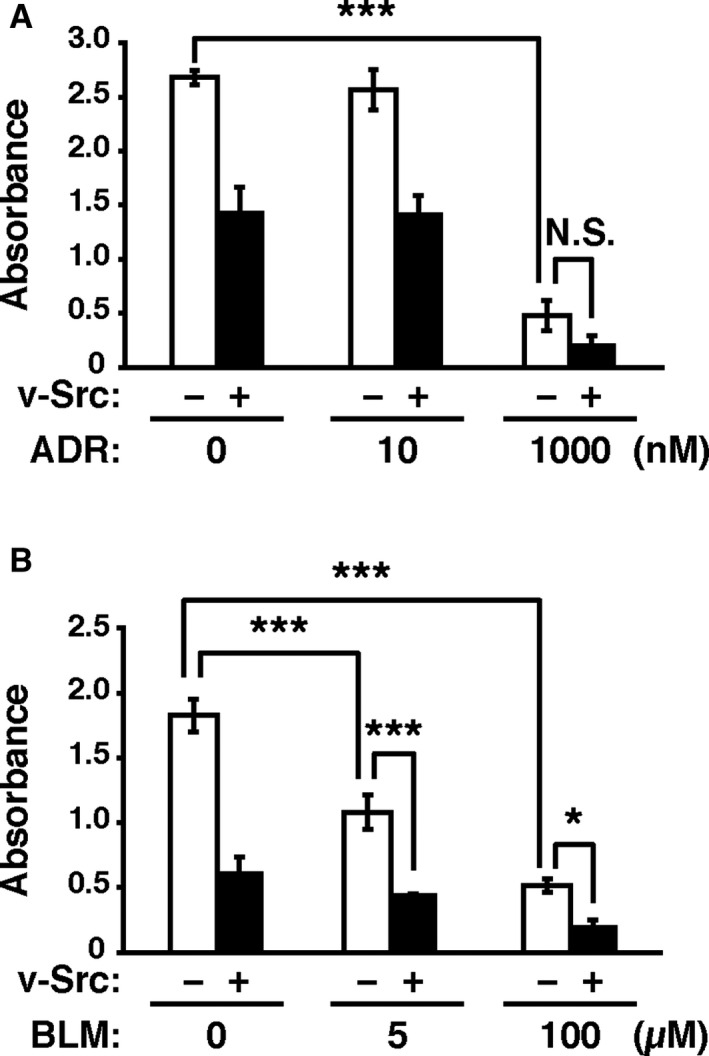
Effects of v‐Src induction on adriamycin and bleomycin‐induced reduction in cancer cell viability. A, B, HeLa S3/v‐Src cells were plated at a density of 3.0 × 10^3^ cells/well of a 96‐well plate and cultured with or without 2 ng/mL Dox in the presence or absence of 10–1000 nM adriamycin (ADR) (A) or 5–100 µM bleomycin (BLM) (B), as shown in Figure 1A. Cell viability was determined by WST‐8 assay 48 hours after removal of indicated drugs. Graphs represent the mean ± SD of three independent experiments. Asterisks indicate significant differences (Tukey's test, ^*^ *P* < .05; ^***^ *P* < .001; N.S., not significant)

Next, we examined whether v‐Src affects the cytotoxicity of anticancer drugs other than MTAs, such as adriamycin and bleomycin. Adriamycin inhibits topoisomerase II, thereby causing double‐strand breaks. Bleomycin induces free‐radical‐mediated DNA single‐ and double‐strand breaks. Consequently, these drugs induce cell death. Actually, adriamycin and bleomycin treatment reduced cell viability in a concentration‐dependent manner (Figure 2A,B). In this experiment, the decreased number of cells (3.0 × 10^3^ cells/well in a 96 well plate) was seeded compared with that used in Figure 1, to obtain the cytotoxic effects of adriamycin and bleomycin. Because this may enhance the v‐Src‐caused suppression of cell proliferation, v‐Src alone decreased the measured absorbance using WST‐8 assay. v‐Src did not restore the cell viability reduced by adriamycin or bleomycin. These results suggest that the suppressive effect of v‐Src on the cytotoxicity of anticancer drugs is specific to MTAs.

### 3.3 Induction of mitotic slippage by v‐Src in microtubule‐targeting agents‐induced mitotic arrest

**FIGURE 3:**
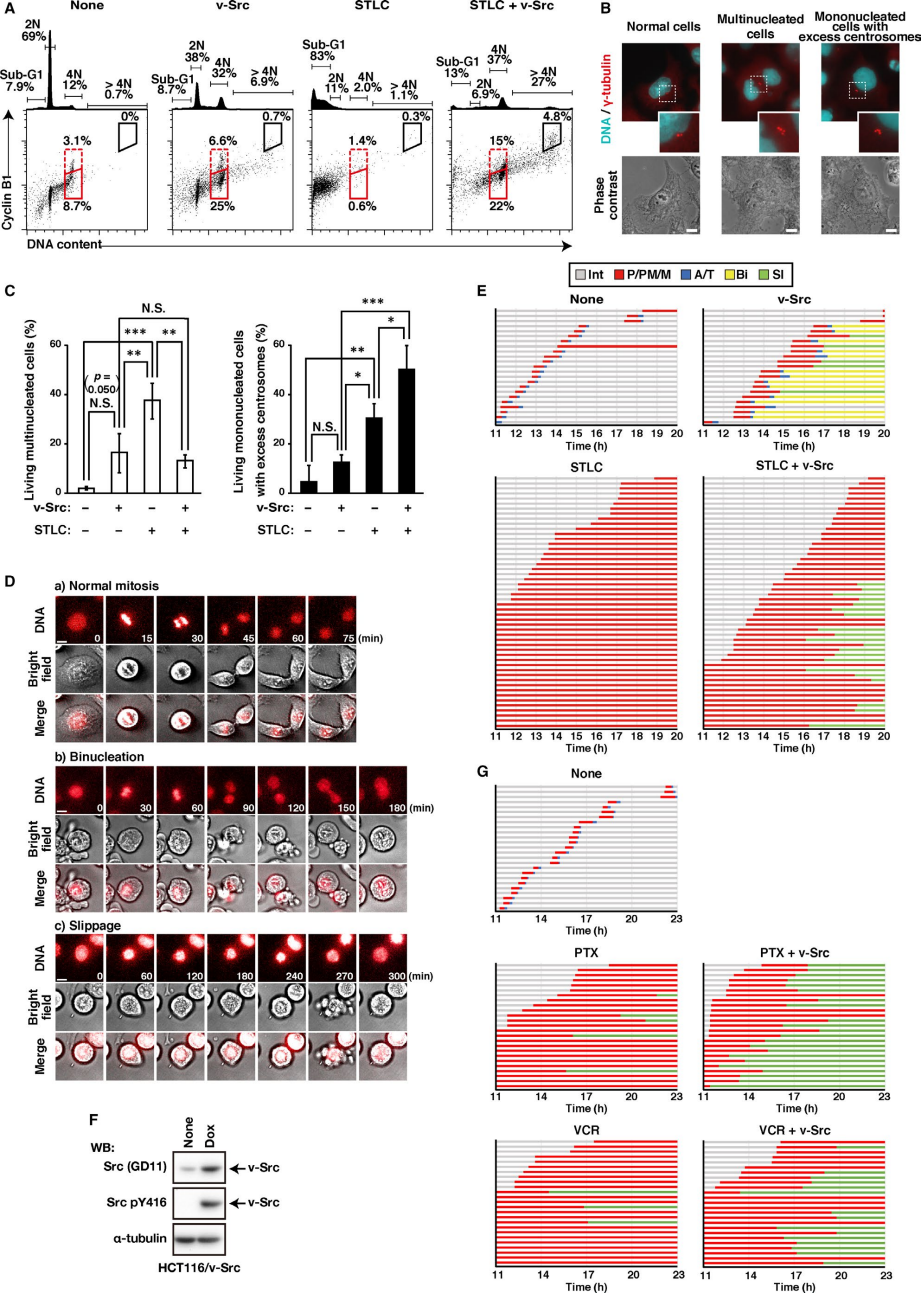
v‐Src‐induced mitotic slippage in microtubule‐targeting agents–treated mitotically arrested cells. A–C, HeLa S3/v‐Src cells were cultured with or without 2 ng/mL Dox in the presence or absence of 20 µM STLC for 24 hours. Then, the cells were washed and further cultured without any drug for 72 hours. A, The cells were fixed, doubly stained with anti‐cyclin B1 and PI, and subsequently analysed by flow cytometry. The bivariate dot plots of cyclin B1 protein level (*y*‐axis, log scale) and DNA content (*x*‐axis, linear scale) are shown together with DNA histograms. The ratios of cells are shown: 4N cells with lower (red solid line) and higher (red dotted line) cyclin B1 levels and 8N cells with higher cyclin B1 levels (black solid line). The ratios of 2N and 4N cells, sub‐G1 cells, and polyploid cells (>4N) are indicated. Each plot represents 4,000 cells. B, C, The cells were fixed and doubly stained with anti‐γ‐tubulin and Hoechst 33342 (DNA staining). B, Representative images were indicated. Magnified images of the areas enclosed by dotted lines are shown. Scale bars, 10 μm. C, (Left) Ratios of living cells having more than two nuclei per single cell were calculated. (Right) Ratios of living cells having more than three γ‐tubulin foci per mononucleated single cell were calculated. Graphs represent the mean ± SD of three independent experiments. Asterisks indicate significant differences (Tukey's test, ^*^ *P* < .05, ^**^ *P* < .01, ^***^ *P* < .001, N.S., not significant). D, E, HeLa S3/v‐Src cells were cultured with or without 2 ng/mL Dox in the presence or absence of 20 µM STLC for 11 hours and then monitored for 9 hours by time‐lapse imaging. D, Representative images of mitotic cells are shown: cells that exhibit normal mitosis (a, normal mitosis), the cleavage furrow regression after furrow ingression (b, binucleation) and the mitotic exit without chromosome segregation (c, slippage). Scale bars, 10 µm. E, The duration of each mitotic phase is shown: prophase/prometaphase/metaphase (P/PM/M: from nuclear envelope breakdown to chromosome alignment; red), anaphase/telophase (A/T: from anaphase onset to cleavage furrow ingression; blue), binucleation (Bi: binucleated interphase cells; yellow) and slippage (Sl: interphase cells that undergo mitotic slippage; green). In total, 23–50 mitotic cells were examined. Several cells were already arrested in mitosis at the beginning of time‐lapse imaging: 24 cells (STLC) and 13 cells (STLC + v‐Src). F, HCT116/v‐Src cells were cultured with or without 1 ng/mL Dox for 24 hours. Whole cell lysates were analysed by Western blotting using anti‐Src, anti‐active Src (Src pY416) and anti‐α‐tubulin antibodies. Full blots are shown in Figure S2B. G, HCT116/v‐Src cells were cultured with or without 1 ng/mL Dox in the presence or absence of 0.1 µg/mL paclitaxel (PTX) or 0.1 µM vincristine (VCR) for 11 hours and then monitored for 12 hours by time‐lapse imaging. The duration of each mitotic phase is shown. In total, 25 mitotic cells were examined. Several cells were already arrested in mitosis at the beginning of the time‐lapse imaging: 12 cells (PTX), 10 cells (PTX + v‐Src), 15 cells (VCR) and 15 cells (VCR + v‐Src)

Our previous work showed that v‐Src causes cytokinesis failure and mitotic slippage, generating tetraploid cells.^7^, ^10^ To investigate whether v‐Src brought about mitotic slippage in STLC‐treated mitotically arrested cells, we first determined the DNA contents and cyclin B1 levels by flow cytometry. The cyclin B1 protein level is gradually increased from G2 phase to M phase, leading to complex formation with CDK1 kinase and its activation,^19^ which is necessary for the mitotic entry. For the mitotic exit, cyclin B1 should be degraded, thereby inactivating CDK1 activity. When cells undergo abnormal mitosis, such as cytokinesis failure and mitotic slippage, and resume the next cell cycle, these tetraploid cells exhibit a 4N DNA content with lower cyclin B1 level in G1 phase (4N‐G1). As shown in Figure 3A, in controls cells (None), cyclin B1 level was increased in 4N cells compared with 2N cells. The ratio of 4N cells with lower cyclin B1 levels was increased upon v‐Src induction (25%, v‐Src), suggesting that tetraploid cells are increased as previously described.^7^ The ratio of 4N cells with lower cyclin B1 levels was strongly increased upon v‐Src induction together with STLC treatment (22%, STLC + v‐Src) similarly to Dox treatment alone, even when the cells underwent mitotic arrest by STLC treatment. Interestingly, this treatment strongly increased the ratio of cells having >4N cells (27%) and cells having 8N DNA content with higher cyclin B1 levels (4.8%), suggesting a possibility that the v‐Src upon STLC treatment generates proliferating tetraploid cells through mitotic slippage and cytokinesis failure.

In v‐Src‐induced mitotic slippage, mitotic chromosomes are de‐condensed without neither chromosome alignment nor chromosome segregation, thereby generating mononucleated cells.^10^ On the other hand, in v‐Src‐induced cytokinesis failure, cleavage furrow is ingressed but finally regressed, generating binucleated cells.^7^ These cells are likely to have excess centrosomes. Therefore, we stained the v‐Src‐expressing and STLC‐treated cells for DNA and the centrosome component γ‐tubulin. v‐Src slightly increased the ratio of multinucleated cells and mononucleated cells having excess centrosomes, whereas STLC treatment alone increased these ratios more (Figure 3C). It is supposed that STLC treatment alone may generate multinucleated cells by chromosome segregation with incomplete mitotic spindle despite presence of STLC and subsequent cytokinesis failure and that a comparable number of cells may undergo mitotic slippage without chromosome segregation. v‐Src expression in addition to STLC treatment increased the ratio of excess centrosome‐containing mononucleated cells and decreased the ratio of multinucleated cells. This suggests that cells overcome the STLC‐mediated mitotic arrest and undergo mitotic slippage.

Furthermore, to determine whether v‐Src actually induced mitotic slippage in STLC‐treated cells, we performed time‐lapse imaging after 11‐hours treatment with Dox and STLC. In binucleation, although the condensed chromosomes were aligned (60 minutes) and segregated (90 minutes), the cleavage furrow was regressed after complete ingression (Figure 3D, panel B). In cells undergoing mitotic slippage, condensed chromosomes were de‐condensed without their alignment and segregation after prolonged mitosis (Figure 3D, panel c). Compared with controls cells whose average duration of mitosis is 41 minutes (Figure 3E: None), v‐Src induction prolonged mitosis (99 minutes) with binucleation and mitotic slippage (Figure 3E: v‐Src), as previously described.^10^ STLC treatment almost completely induced mitotic arrest in the prometaphase‐like state within the analysis (Figure 3E: STLC). STLC treatment of v‐Src‐expressing cells caused mitotic arrest; however, 53% of the cell population underwent mitotic slippage after the arrest (Figure 3E: STLC + v‐Src). Moreover, to examine whether this slippage was induced in the other cell line, we used HCT116 colorectal cancer cells capable of inducible expression of v‐Src by Dox^7^ (Figure 3F). Although paclitaxel or vincristine treatment clearly induced mitotic arrest (Figure 3G: PTX, VCR), v‐Src induced mitotic slippage in 88% of paclitaxel‐treated or 60% of vincristine‐treated mitotically arrested cells (Figure 3G: PTX + v‐Src, VCR + v‐Src), suggesting that v‐Src induces mitotic slippage in mitotically arrested cells. Taken together, these results suggest that v‐Src‐induced mitotic slippage causes the reduction of sensitivity of v‐Src‐expressing cells to MTAs.

### 3.4 Effects of PLK1‐ and Aurora kinase inhibitors on v‐Src‐expressing cells

**FIGURE 4:**
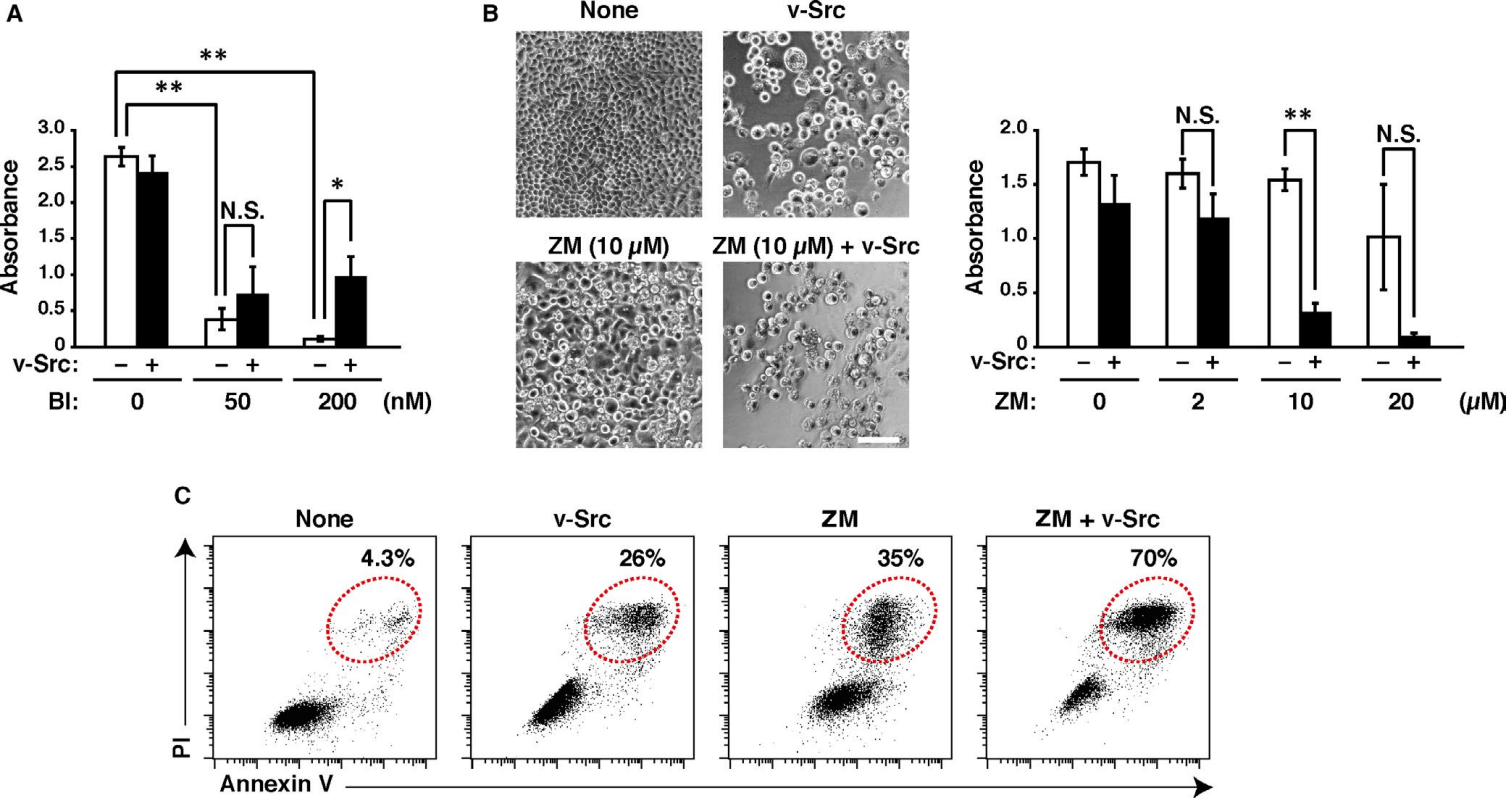
The Aurora kinase inhibitor ZM447439 induces cell death in v‐Src‐expressing cells. A, B, HeLa S3/v‐Src cells were plated at a density of 8.0 × 10^3^ cells/well of a 96‐well plate and cultured with or without 2 ng/mL Dox in the presence or absence of inhibitors [50–200 nM BI2536 (BI) (A) or 2–20 µM ZM447439 (ZM) (B)], as shown in Figure 1A. Cell viability was determined by WST‐8 assay 48 hours after removal of the indicated drugs. Graphs represent the mean ± SD of three independent experiments. Asterisks indicate significant differences (Games–Howell test, ^*^ *P* < .05; ^**^ *P* < .01; N.S., not significant). B, (Left) Phase‐contrast images were obtained 48 hours after the indicated drugs removal. Scale bar, 50 µm. C, HeLa S3/v‐Src cells were cultured with or without 2 ng/mL Dox in the presence or absence of 10 µM ZM for 24 hours. Then, the cells were washed and further cultured without any drug for 48 hours. The cells were stained with Annexin V and PI as described under ‘MATERIALS AND METHODS’ and analysed by flow cytometry. The bivariate dot plots of Annexin V level (*y*‐axis, log scale) and PI level (*x*‐axis, log scale) are shown. The regions designated by red dotted lines include Annexin V‐positive / PI‐positive cells, and the ratios within the region are shown. Each plot represents 25,000 cells

PLK1 and Aurora kinases are essential for proper mitotic progression, and those expressions are increased in proliferating cancer cells.^20^ Given that those inhibitors reduce the cancer cell viability, PLK1 and Aurora kinases are prominent targets for new anticancer drugs.^21^, ^22^ Thus, we examined whether v‐Src affected the cytotoxicity of PLK1 and Aurora kinase inhibitors. Although treatment with the PLK1 inhibitor BI2536 reduced cell viability in a dose‐dependent manner (Figure 4A), v‐Src slightly restored the cell viability reduced by BI2536, similar to its effects on the paclitaxel‐ and vincristine‐reduced cell viability. BI2536 treatment for 24 hours caused mitotic arrest (Figure S1A), suggesting a possibility that v‐Src overrides BI2536‐induced mitotic arrest and suppresses the reduction in cell viability. Next, we treated cells with the Aurora kinase inhibitor ZM447439 with or without Dox. ZM447439 treatment at a high dose reduced the cell viability (Figure 4B). Intriguingly, ZM447439 treatment strongly reduced cell viability in v‐Src‐expressing cells, even when the 10 µM ZM447439 treatment alone did not reduce the cell viability. Moreover, flow cytometry analysis showed that ZM447439 treatment strongly increased the ratio of Annexin V‐positive / PI‐positive dead cells in v‐Src‐expressing cells (Figure 4C). These results suggest that Aurora kinase inhibition exerts a cytotoxic effect against v‐Src‐expressing cancer cells.

## 4 DISCUSSION

Here, we show that v‐Src restores cancer cell viability that is reduced by the microtubule‐targeting drugs STLC, paclitaxel and vincristine. Similarly, v‐Src slightly restores cancer cell viability in cells treated with an inhibitor of PLK1, a core mitotic regulator. On the other hand, this does not occur in cells treated with adriamycin and bleomycin, which are not anti‐mitotic drugs. Time‐lapse imaging clearly shows that v‐Src causes mitotic slippage upon MTAs‐induced mitotic arrest, thereby increasing the ratio of mononucleated cells with excess centrosomes, but not that of multinucleated cells. Similar to v‐Src induction, the MPS1 inhibitor restores cell viability reduced by STLC, suggesting that v‐Src‐mediated override of SAC leads to cell survival. Interestingly, an inhibitor of Aurora kinase, another core mitotic regulator, causes strong cell death and drastically reduces the cell viability of v‐Src‐expressing cells. Therefore, to effectively use anticancer drugs targeting mitosis, Src activity in cancer cells should be taken into consideration.

We previously reported that v‐Src causes mitotic defects, such as chromosome bridge formation, cytokinesis failure and mitotic slippage.^7^, ^9^, ^10^ Chromosome bridge formation contributes to aneuploidy and micronucleus formation. Cytokinesis failure and mitotic slippage lead to tetraploid cell formation. Considering the effects of v‐Src on mitosis, we demonstrated that v‐Src‐induced mitotic abnormalities may cause stochastic oncogenic transformation.^8^ Here, we found a novel aspect of v‐Src on anticancer drugs: v‐Src causes mitotic slippage in various MTAs‐treated mitotically arrested cells, thus attenuating those cytotoxicities. Mitotic slippage upon MTA treatment gives rise to acquiring excess centrosomes and DNA contents in v‐Src‐expressing cells. Even when cells acquire excess number of centrosomes, those cells can form bipolar spindles by the clustering of centrosomes; however, this cell division is prone to result in asymmetrical chromosome segregation.^23^ This raises the risk that MTA treatment of cancer cells expressing highly activated Src may lead to cancer malignancy through chromosome instability.

v‐Src is the constitutive active mutant of cellular counterpart c‐Src.^1^, ^3^, ^4^ c‐Src also contributes to tumorigenesis in various tissues by up‐regulating its protein level or kinase activity.^24^, ^25^ c‐Src is also known to decrease the sensitivity of cancer cells to anticancer drugs, including paclitaxel. c‐Src activates PI3K‐AKT, STAT3/5 and anti‐apoptotic proteins, thereby attenuating the cytotoxic effect of paclitaxel.^26^ Considering the present study of v‐Src effect on MTAs, it is possible that highly activated c‐Src may also cause mitotic slippage. We recently reported that knockdown of Csk, an inhibitory kinase of Src, slightly but significantly induces mitotic slippage.^10^ Therefore, highly activated c‐Src may contribute to the suppression of cytotoxic effects of MTAs, leading to cancer malignancy at the worst case.

v‐Src directly phosphorylates Tyr‐15 of CDK1, whose phosphorylation inhibits its kinase activity, leading to mitotic slippage through CDK1 inactivation after mitotic entry.^10^ Here, we used the Eg5 inhibitor STLC, the microtubule stabilizer paclitaxel and the microtubule‐depolymerizing drug vincristine. The common consequence among these agents is mitotic arrest due to SAC activation. Prolonged mitosis gradually increases the apoptotic signal, thereby causing cell death.^27^ Mitotic slippage, another fate during mitotic arrest, is thought to be an evasive way to survive from the mitotic arrest.^28^ Indeed, our previous work showed that CDK1 inactivation induced mitotic slippage in cells mitotically arrested by STLC, thereby reducing the cytotoxicity of STLC.^10^ Although MTAs caused cell death and reduced cell viability, v‐Src suppressed that cytotoxicity (Figure 1). Furthermore, time‐lapse imaging analysis showed that MTAs cause strong mitotic arrest without chromosome alignment; however, v‐Src induces mitotic slippage in more than half of cells after mitotic arrest within the analysis (Figure 3E,G). Therefore, v‐Src‐mediated CDK1 inactivation contributes to mitotic slippage in MTAs‐treated mitotically arrested cells, thus reducing MTA cytotoxicity and promoting cancer cell survival.

Recent reports showed that many cancer cells show tetraploid or near‐tetraploid phenotypes,^29^ and these characteristics generally link to tumour malignancy and unfavourable prognosis in patients.^30^, ^31^ Tetraploid cell formation is accompanied by centrosome duplication, and excess centrosome‐mediated multipolarity gives rise to chromosome instability through asymmetrical chromosome segregation.^32^ Tetraploid cells are generated by mitotic defects, such as mitotic slippage and cytokinesis failure; anti‐mitotic drugs also contribute to generate those cells.^33^ It is known that tetraploid cells undergo cell cycle arrest at G1 phase by the tetraploidy checkpoint,^34^ which prohibits cells from entering the next cell division cycle. In tetraploidy checkpoint activation, excess centrosomes and cytoskeleton abnormalities trigger Rac activation, thereby activating the tumour suppressor LATS2, which is a Hippo pathway regulator. Activated LATS2 sequestrates nuclear YAP in the cytoplasm and activates p53 by Mdm2 inactivation.^35^ Our recent research showed that v‐Src inactivates tetraploidy checkpoint, leading to nuclear accumulation of YAP in multinucleated cells.^36^ In the present study, flow cytometry analysis showed that cells having 8N DNA content and higher cyclin B levels were observed when v‐Src‐expressing cells were treated with STLC, suggesting a possibility that v‐Src‐expressing tetraploid cells can evade from tetraploidy checkpoint and continue to proliferate. MTAs might induce chromosome instability and lead to tumour malignancy in cancer cells expressing Src with high activity.

We found that the Aurora kinase inhibitor induced severe cell death in v‐Src‐expressing cells (Figure 4B,C). What is the mechanism of cell death promotion by v‐Src in the Aurora kinase inhibitor‐treated cells? The Aurora kinase inhibitor ZM447439 inhibits the kinase activities of Aurora A and Aurora B kinases at a higher concentration.^37^ Aurora A localizes to the centrosome and exerts chromosome congression.^38^ Kinetochore‐localized Aurora B activates SAC while all kinetochores are not properly attached to microtubules, and midzone‐localized Aurora B regulates cleavage furrow ingression to complete the cell division.^39^ Although chromosome congression is attenuated in ZM447439‐treated cells, mitosis progresses due to SAC inactivation. Furthermore, the cleavage furrow cannot be formed, resulting in cytokinesis failure and generation of binucleated cells.^37^, ^40^ Indeed, time‐lapse imaging revealed that ZM447439‐treated cells progressed mitosis without mitotic arrest and cleavage furrow ingression, resulting in binucleation (Figure S1B). Given that v‐Src causes Aurora B delocalization from the spindle midzone,^7^ cytokinesis failure may be strongly induced by ZM447439 treatment of v‐Src‐expressing cells. It was reported that ZM447439 generates polyploid cells and causes apoptosis through the mitochondria pathway.^41^ Furthermore, v‐Src is known to generate mitochondria‐dependent apoptotic signals,^42^ and overexpression of mitochondria‐localized c‐Src reduces the cell viability through reduction of mitochondria activity by phosphorylation of mitochondrial single‐stranded DNA‐binding protein.^43^ These studies suggest a possibility that v‐Src induction together with ZM447439 treatment may accelerate apoptotic signalling in the mitochondria of polyploid cells, leading to severe cell death.

Recent reports showed that oncogenic events, such as KRAS mutation and MYC overexpression, causes mitotic defects, and oncogene‐induced chromosome instability is thought to be attributed to those mitotic abnormalities.^44^, ^45^ Our reports regarding v‐Src oncogene also supports the idea that there is a relationship between oncogenic mitotic abnormalities and genome instability. Oncogenes frequently confer resistance to anticancer drugs through several ways in cancer cells. Similar to v‐Src that causes mitotic slippage to evade from mitotic arrest caused by various MTAs, it is possible that other oncogenes may induce mitotic slippage as a different way of acquiring resistance against anticancer drugs.

In conclusion, we show that v‐Src oncogene causes mitotic slippage in MTAs‐treated mitotically arrested cells, thereby suppressing the cytotoxicity of those agents. The resulting tetraploid cells with excess number of centrosomes have a potential to induce structural and numerical chromosome instability, increasing cancer cell malignancy through stochastic genetic alterations.

## CONFLICT OF INTEREST

There are no conflicts of interest.

## Author Contribution

**Ryuzaburo Yuki:** Conceptualization (equal); Data curation (equal); Formal analysis (equal); Investigation (equal); Methodology (equal); Project administration (equal); Validation (equal); Visualization (equal); Writing‐original draft (lead); Writing‐review & editing (supporting). **Mari Hagino:** Conceptualization (supporting); Data curation (equal); Formal analysis (equal); Methodology (equal); Validation (equal). **Sachi Ueno:** Formal analysis (supporting); Investigation (supporting). **Takahisa Kuga:** Conceptualization (equal); Supervision (supporting). **Youhei Saito:** Supervision (supporting). **Yasunori Fukumoto:** Resources (supporting); Supervision (supporting). **Noritaka Yamaguchi:** Resources (supporting); Supervision (supporting). **Naoto Yamaguchi:** Resources (equal); Supervision (supporting). **Yuji Nakayama:** Conceptualization (lead); Data curation (equal); Funding acquisition (lead); Investigation (supporting); Methodology (equal); Project administration (lead); Resources (equal); Supervision (lead); Validation (supporting); Visualization (lead); Writing‐original draft (supporting); Writing‐review & editing (lead).

## Supporting information

Supplementary Material

## Data Availability

The data used to support findings of the study are available from the corresponding author upon reasonable request.
