# Supplementary material for: The tyrosine kinase v‐Src modifies cytotoxicities of anticancer drugs targeting cell division
Source: J Cell Mol Med. 2021 Jan 19;25(3):1677–87. doi: 10.1111/jcmm.16270 (PMC7875926; doi:10.1111/jcmm.16270)
Supplement: Supplementary file 1 — Supplementary Material [file JCMM-25-1677-s001.pdf]

Supplementary figures

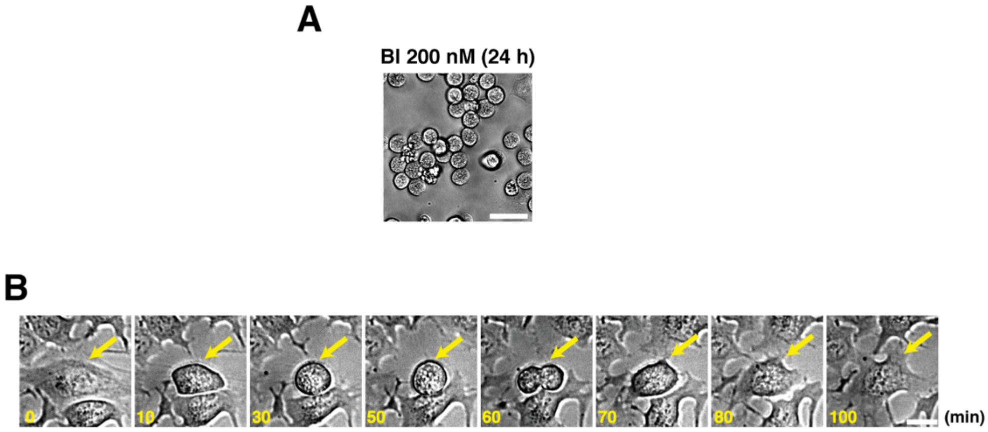

Supplementary Figure 1. Representative images of BI2156- or ZM447439-treated HeLa S3/v-Src cells.

(A) HeLa S3/v-Src cells were cultured with 200 nM BI2156 (BI) for 24 h, and a phase-contrast image was obtained. Scale bar, 50 µm. **(B)** HeLa S3/v-Src cells were cultured with 10 µM ZM447439, and the time-lapse imaging was started. Selected frames from the phase-contrast images, which were obtained 7 h after ZM447439 treatment, are shown. Arrows indicate cell whose cleavage furrow was ingressed and finally regressed. Scale bar, 20 µm.

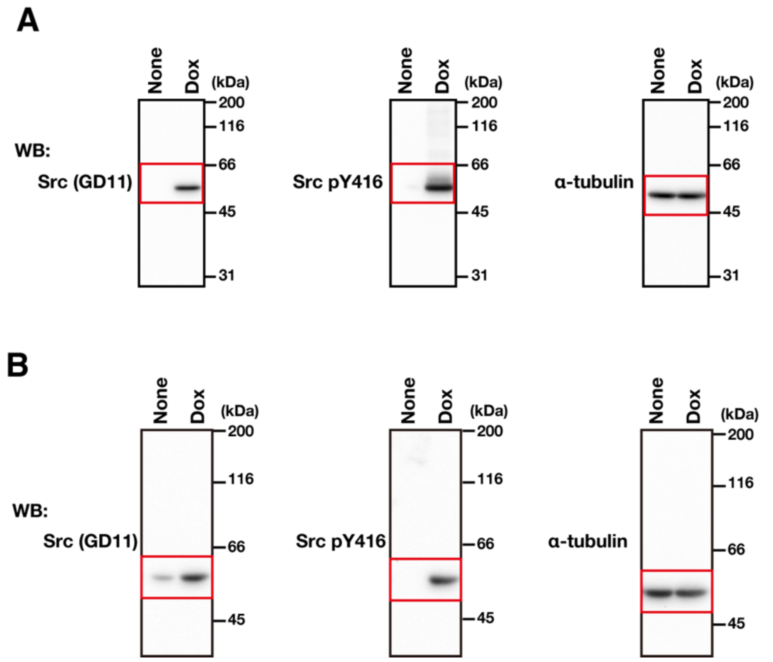

Supplementary Figure 2.   Full-length blots for Figure 1B (A) and Figure 3F (B).
